# Assessment of International Consensus Group for Haematology smear review rules among patients with *Plasmodium falciparum* malaria in Johannesburg, South Africa

**DOI:** 10.4102/ajlm.v7i1.715

**Published:** 2018-11-29

**Authors:** Jenifer L. Vaughan, Nazeer Alli, Sandra Havyarimana, Estee Benade

**Affiliations:** 1 Department of Molecular Medicine and Haematology, University of the Witwatersrand, Johannesburg, Gauteng, South Africa; 2 Department of Haematology, Chris Hani Baragwanath Academic Hospital, Johannesburg, Gauteng, South Africa; 3 Excellent Medical Laboratory Services, Oshakati, Oshana, Namibia

## Abstract

A peripheral blood smear review is a useful but labour intensive adjunct to the full blood count and differential count. In this study, we retrospectively evaluated the performance of the International Consensus Group for Haematology smear review rules for detection of malaria in 153 samples with *Plasmodium falciparum* parasitaemia. Review rules were triggered in 132 (86.3%) samples, including all patients with severe malaria. Of the 21 false negative samples, 14 (66.7%) had haemoglobin values ≥ 10g/dl and platelets > 120x10^9^/l.

## Introduction

Malaria affects in excess of 200 million people annually, with 90% of cases occurring in Africa.^1^ It has a significant mortality rate, with 445 000 malaria-related deaths documented by the World Health Organization in 2016.^1^ Its diagnosis rests on rapid diagnostic tests, which detect malaria antigen, as well as microscopic identification of parasites on thick and thin smear reviews (SR).^1,2^ However, a proportion of patients are diagnosed incidentally where malaria is not suspected clinically following a peripheral blood SR from a sample submitted for a routine full blood count (FBC) or differential white cell count (DWCC). As such, ensuring the maximum possible pick-up rate of malaria in samples submitted for a FBC or DWCC is a priority. However, perpheral blood SR is labour intensive and requires skilled morphologists, which are in short supply. Careful selection of samples for SR is therefore vital. This was recognised by the International Consensus Group for Haematology (ICGH), who compiled a set of evidence-based guidelines directing SR in order to facilitate detection of clinically important morphologic features while reducing the number of SR done.^3^ These rules have reduced SR rates in many laboratories, but adjustment is often required to accommodate for local pathology patterns, clinician preferences and resource availability. For instance, in laboratories servicing haematology-oncology centres, increased vigilance for the detection of neoplastic cells in the blood is required. Many clinical haematologists prefer manual review of all samples collected from patients known to have haematological diseases, but this is not always feasible in laboratories with staffing constraints. Laboratory-specific adjustment of the ICHG SR guidelines should therefore be undertaken where necessary, with ongoing monitoring in order to facilitate further fine-tuning of the rules and to reduce false negative rates where possible. In particular, every effort should be made to ensure that life-threatening pathologies (including malaria, haemolysis, megaloblastosis and haematologic malignancies) are not missed as a result of implementation of such rules, and where pathology has been found to have been overlooked, adjustments to the SR rules should be considered.

At the Chris Hani Baragwanath Academic Hospital National Health Laboratory Service laboratory in Johannesburg, South Africa, we validated these rules, and made necessary adjustments to accommodate local peculiarities (Table 1). For example, leukopenia is very common in our setting due to the combined effects of benign ethnic neutropenia (which affects up to 60% of black South Africans)^4^ and frequent HIV infection (> 1/3 of the FBCs processed in our laboratory are collected from HIV-positive patients).^5^ Consequently, the low white cell count threshold for smear review was reduced from 4 × 10^9^/l to 2 × 10^9^/l. Other changes were made on the basis of manufacturer recommendations (such as review with an immature granulocyte count > 5% as compared to the presence of an immature granulocyte flag +), acceptable false positive rates in our experience (such as nucleated red blood cells > 5/100 white cells as opposed to the presence of a nucleated red blood cell flag +) or to enhance the clinical significance of findings (such as SR when the analyser flags possible platelet clumping only when the platelet count is low as opposed to the presence of a platelet clumping flag with any platelet count). These rules were validated in a representative selection of samples, and shown to have an acceptable false negative rate (< 5%). However, the number of samples collected from patients with malaria for the validation process was limited. This study therefore aimed to retrospectively validate the performance of the Chris Hani Baragwanath Academic Hospital National Health Laboratory Service SR rules in the detection of this critical pathology.

**TABLE 1:** Modified ICGH rules implemented at the Chris Hani Baragwanath Academic Hospital National Health Laboratory Service laboratory.

Parameter flags	Analyser suspect flags
WCC > 30 OR < 2 × 109/l† AND first time	?Red cell fragment flag AND Hb < 10 g/dl†
Hb < 7 OR > 2 g/dl above the reference interval AND first time	?Plt clumping AND Plts < 120 × 109/l†
MCV > 105 OR < 75 fL AND first time	?Atypical lymphocytes
RDW > 22% AND first time	?Blasts
Plts < 100 or >1000 × 10^9^/l AND first time OR Delta check failed	Pathological left shift (Immature granulocytes > 5%)
Incomplete or failed diff count	Dysplasia indications†
Neutrophils‡ < 1 × 10^9^/l AND first time	-
Lymphocytes‡ > 5 × 109/l (adult) or > 7 × 109/l (< 12 years) AND first time	-
Monocytes‡ > 1.5 × 109/l (adult) or > 3 × 109/l (< 12 years) AND first time	-
Eosinophils > 2 × 109/l AND first time	-
Basophils > 0.5 × 109/l AND first time	-
Immature granulocytes > 5%†	-
NRBCs > 5/100 white cells? AND first time	-
Reticulocytes‡ > 0.1 × 1012/l AND first time	-

Hb, Haemoglobin; WCC, white cell count; MCV, mean cell volume; RDW, red cell distribution width; Plt, platelets; NRBC, nucleated red blood cells, ICGH, International Consensus Group for Haematology.

† , Rules that differ from the ICGH rules.

‡ , Refers to absolute counts.

## Materials and methods

### Ethical considerations

The study was approved by the Human Research Ethics Committee of the University of the Witwatersrand, Johannesburg (ref. no. M150160).

### Study design

Samples requested for malaria testing (*Plasmodium falciparum* antigen along with thick and thin SR) between January and April of 2015 were extracted from the laboratory information system (TrakCare, InterSystems, Cambridge, Massachusetts, United States) and those with positive test results identified. Samples positive for the *P. falciparum* antigen but negative on microscopy were excluded. The laboratory information system was then searched for FBCs performed on the same day as the malaria test in order to evaluate whether SR rules were triggered. FBC samples were processed using a Sysmex XE-5000 analyser and Sysmex Information System middleware (Sysmex, Kobe, Japan). All ICGH rules were applied to samples with both a FBC and DWCC requested, whereas only parameter-related rules were applied to samples with requests for a FBC alone. SR was also performed in samples when the analyser’s inbuilt malaria suspect flag was triggered. The rules violated for each FBC identified were documented, and the performance of the rules assessed. As a retrospective, laboratory-based study, no clinical information was available. Determination of which patients had severe malaria was therefore defined on the basis of laboratory criteria, viz. severe anaemia (Hb < 7 g/dl in adults and < 5 g/dl in children aged < 12 years), parasitaemia > 10%, renal failure (urea > 20 mmol/l or creatinine > 265 umol/l) hypoglycaemia (< 2.2 mmol/l) or hyperbilirubinaemia (> 50 umol/l with > 100 000 parasites/ul).^6^ Patients admitted to the intensive care unit were also classified as having severe malaria.

### Statistical analysis

Continuous data are presented as the median (interquartile range) and categorical data as frequencies and percentages. The Mann-Whitney *U*-test was used to compare variables of interest (both ordinal and continuous). The false negative rate was calculated as the proportion of all patients with malaria where SR rules were not triggered. Statistical analysis was performed using STATISTICA software, version 12.0 (Stat Soft (Pty) Ltd, Tulsa, Oklahoma, United States). Statistical significance was accepted at a two-sided *p*-value of 0.05.

## Results

A total of 153 samples collected from 130 patients met the inclusion criteria. Pertinent demographic information is summarised in Table 2. Most patients had only a FBC performed, with a DWCC being requested in 63 (41.2%) patients. SR rules were triggered in 132 (86.3%) cases, either in the current specimen or in previously submitted samples. Of the reviewed samples 63 were submitted for a DWCC (9/63 in previous and 54/63 in current samples) and 69 for a FBC only (16/69 in previous and 53/69 in current samples). The rule trigger rate did not differ between samples requested for a DWCC as compared to those requested only for a FBC (85.7% vs 86.7%; *p* = 0.87), but was significantly higher among patients classified as having severe malaria as compared to those without severe malaria (100.0% vs 81.9%, *p* = 0.017).

**TABLE 2:** Pertinent demographic data.

Age (years) (Median [IQR])	23.5 (6.3–32.0)
Male:Female ratio	1.52:1
Parasitaemia (%) (Median [IQR])	2.12 (0.5–3.4)
Severe malaria (*n*[%])	37 (24.2)
Platelets (×10^9^/l) (Median [IQR])	77 (54.0–115.0)
Hb (g/dl) (Median [IQR])	10.8 (7.7–12.3)
Thrombocytopenia (*n*[%])†	130 (85.0)
Anaemia (*n*[%])‡	127 (83.0)

Hb, Haemoglobin; IQR, interquartile range.

† , < 150 × 10^9^/l

‡ , Haemoglobin below the age-specific reference interval (< 10.5 g/dl age 6 months–2 years, < 10.8 g/dl age 2–3 years, < 11.1 g/dl age 3–5 years, < 10.7 g/dl age 5–8 years, < 10.3 age 8–13 years, < 12.1 g/dl adult females, < 14.3 g/dl adult males).

### Smear review rules triggered

Parameter rules were triggered in all samples, the commonest being the thrombocytopenia rule, which was flagged in 105 patients (Table 3). Common analyser morphology flags included those querying the presence of atypical lymphocytes, immature granulocytes and blasts, but these were not triggered in the absence of parameter flags.

**TABLE 3:** Parameter rules triggered among patients with malaria.

Parameter	*n*	%
Platelets < 100 × 10^9^/l	105	68.6
Hb < 7 g/dl	24	15.7
MCV < 75 fl	40†	26.8
MCV > 105 fl	1†	0.7
RDW > 22%	6†	4.0
Reticulocytes > 0.1 × 10^12^/l	4‡	40
WCC < 2 × 10^9^/l (1st time)	2	1.3
Neutrophils < 1 × 10^9^/l (1st time)	2†	3.2
Monocytes > 1.5 × 10^9^/l (adults) OR > 3 × 10^9^/l (< 12 years)	3§	4.8
Lymphocytes > 5 × 10^9^/l (adults) OR > 7 × 10^9^/l (< 12 years)	1	1.6

Hb, Haemoglobin; WCC, white cell count; MCV, mean cell volume; RDW, red cell distribution width; Plt, platelets. *n*, the number of all samples with the parameter of interest. The total number of all samples for each parameter is 153 unless indicated otherwise in the key; %, Percentage.

† , *N* = 149

‡ , *N* = 10

§ , *N* = 63

### Analysis of patients without smear review rules triggered

Among the 21 patients in whom no SR rules were triggered, 19 (90.5%) had a haemoglobin level ≥ 10 g/dl, 14 (66.7%) had a platelet count >120 × 10^9^/l and 8 (38.1%) had a platelet count >150 × 10^9^/l. The malaria parasitaemia did not differ statistically between those with and without SR rules triggered (median 1.9% vs 2.1%; *p* = 0.44), and was < 0.5% in six patients. Nine (42.9%) of the samples with no SR rules triggered a request for a DWCC, and all were the first or only sample submitted.

### Assessment of morphology findings

When SR was performed, malaria parasites were missed in 13.0% of cases, predominantly when the parasitaemia was low (median parasitaemia was 0.35% in those where parasites were missed vs 3.1% in those with parasites identified). The parasite detection rate was significantly greater in patients with a parasitaemia > 0.5% as compared to those with lower parasite loads (93.1% vs 37.5%; *p* < 0.0001).

## Discussion

In this retrospective study assessing the performance of modified ICGH SR rules in patients with malaria, SR was prompted in 86.3% of cases. Parameter flags were triggered in all patients, the commonest being the thrombocytopenia flag which was triggered in 68.6% of cases. The false negative rate (12.7%) was higher than that recommended by the ICGH (< 5%),^3^ largely due to the presence of near normal blood counts (haemoglobin > 10 g/dl and platelets >120 × 10^9^/l) in 70% of patients with no SR rules violated. Analyser morphology flags were evaluated only among samples with a request for a DWCC, but these did not make a substantial contribution to increasing the SR rate, with no statistical difference between those with and without a DWCC requested (85.7% vs 86.7%; *p* = 0.87). Clearly, a proportion of cases with malaria will inevitably be missed using current SR rules. Reassuringly, SR rules were triggered in all patients with severe malaria, thus reducing the likelihood of this condition being missed when not suspected clinically. However, a substantial proportion (13.0%) of cases were missed upon SR, most commonly in cases with very low paraistaemia (< 0.5%). This highlights the poor sensitivity of microscopy for malaria diagnosis, which is highly dependent on operator skill and further compromised by the pressures of large caseloads.^7^ Alternate means of screening blood samples for malaria (where specific malaria testing has not been requested) would be of value. Malaria detection technologies offered by several haematology analysers are of interest in this respect, as a FBC is invariably requested in hospitalised patients with fever of uncertain origin. In Sysmex analysers, malaria suspect flags are generated on the basis of ‘pseudo-eosinophilia’ (which detects pigment-laden granulocytes or monocytes) or abnormalities of the DWCC and reticulocyte scatterplots. The reported sensitivity of these parameters is variable (46.2% – 69.4% for the abnormal DIFF scattergram or pseudo-esoinophilia,^8,9^ 60% – 97% for abnormalities of the WBC/BASO scatterplot^10^ and 77% for abnormalities of the reticulocyte scattergram^10^), with superior performance among patients with *P. vivax* infection.^10,11^ In this study, a malaria flag was not triggered by the middleware in any of the cases, most likely due to the rarity of *P. vivax* infection in Johannesburg. This mirrors the poor performance of these parameters in patients with *P. falciparum* malaria reported elsewhere^10,11^ and suggests that the currently available malaria-specific Sysmex flags are not of significant utility in our setting. Two algorithm-based approaches for Sysmex analysers were developed by Campuzano-Zuluaga et al. using novel and research-based parameters (such as an increase in the number of events detected in the LYMPH-y channel and the number of events within a currently unevaluated area of the WBC/BASO scatterplot [designated the WBC/BASO {III} counting area]), and showed good diagnostic accuracy (> 85%) for *P. falciparum*.^10^ The utility of one of these algorithms (the N-OD1_Pf_ model) was confirmed by Dubreuil et al., who showed a sensitivity of 77% for *P. falciparum* detection using this algorithm in a non-endemic area (France).^11^ Further assessment of such models in our setting would be of interest. Lastly, the new Sysmex XN-30 haematology analyser has novel malaria detection technology^12^ currently in the testing phase, which reportedly has a sensitivity of 98.4% for *P. falciparum* detection (personal communication Prof. Theresa Coetzer, Dept of Molecular Medicine and Haematology, University of the Witwatersrand, Johannesburg). The study is ongoing but preliminary results are encouraging. However, it remains to be seen whether this technology could be incorporated on existing routine haematology platforms.

Other malaria-specific testing modalities carry superior sensitivity rates to conventional microscopy, such as malaria antigen rapid detection kits and various point-of-care kits employing polymerase chain reaction technology (such as the Illumigene ®, which targets a 214-base-pair segment of a non-coding region of *Plasmodium* species mitochondrial DNA^13,14^ and has a limit of detection for *P. falciparum* and *P. vivax* of 2 and 0.125 parasites/µml, respectively^15^). While such detection rates are impressive, these modalities are not suitable for incidental detection of malaria, but are of primary value when malaria is suspected clinically.

Limitations of this study include the paucity of clinical information, as well as the absence of non-falciparum species among the cases included.

### Conclusion

The international consensus group for haematology smear review rules have a significant false negative rate for the detection of *P. falciparum* malaria, but perform well in patients with severe malaria. Until more sensitive technologies for incidental malaria detection are widely available, clinical vigilance for this condition is required.
